# Circ_0078767 Inhibits the Progression of Non-Small-Cell Lung Cancer by Regulating the GPX3 Expression by Adsorbing miR-665

**DOI:** 10.1155/2022/6361256

**Published:** 2022-03-17

**Authors:** Xiting Liu, Ze Chen, Yuqiang Wu, Feng Gu, Dong Yan, Lei Yang, Qin Ma, Caihong Fu

**Affiliations:** ^1^Department of Respiratory Oncology, Gansu Provincial Cancer Hospital, Lanzhou, Gansu, China; ^2^Department of Radiology, Gansu Provincial Cancer Hospital, Lanzhou, Gansu, China; ^3^Endoscopy Center, Gansu Provincial Cancer Hospital, Lanzhou, Gansu, China

## Abstract

Non-small-cell lung cancer (NSCLC) is one of the most serious cancers. The circular RNA_0078767 (circ_0078767) expression was decreased in NSCLC tissues. However, the molecular mechanism of circ_0078767 remains unknown. The expression of circ_0078767, microRNA-665 (miR-665), and glutathione peroxidase 3 (GPX3) was detected by quantitative real-time fluorescence polymerase chain reaction (qRT-PCR). Cell proliferation, migration, and invasion were detected by colony formation assay and transwell assay, respectively. The lactate production and glucose consumption were tested by glycolysis. Western blot examined the protein levels of hexokinase-2 (HK2), matrix metalloproteinase-9 (MMP9), and GPX3 cells. Circinteractome predicted the relationship between miR-665 and circ_0078767 or GPX3 and was verified by dual luciferase reporter assays. The xenotransplantation model was established to study the role of circ_0078767 in vivo. The expression of circ_0078767 and GPX3 was decreased in NSCLC tissues, while the expression of miR-665 was increased. Circ_0078767 can sponge miR-665, and GPX3 is the target of miR-665. In vitro complement experiments showed that knockdown of circ_0078767 significantly promoted malignant behavior of NSCLC, while cotransfection of miR-665 inhibitor partially reduced this change. In addition, the GPX3 overexpression decreased the promoting effects of miR-665 upregulation on proliferation, migration, and invasion of NSCLC cells. Mechanically, circ_0078767 regulates the GPX3 expression in NSCLC cells by spongy miR-665. In addition, in vivo studies have shown that downregulation of circ_0078767 promotes tumor growth. Circ_0078767 silencing promotes proliferation, migration, invasion, and glycolysis of NSCLC cells by regulating the miR-665/GPX3 axis, suggesting that circ_0078767/miR-665/GPX3 axis may be a potential regulatory mechanism for the treatment of NSCLC.

## 1. Introduction

Lung cancer is one of the most deadly malignancies in the world. Non-small-cell lung cancer (NSCLC) is the most common type of lung cancer, accounting for about 85% of all lung cancers [1, 2]. Although advanced therapies can effectively control many primary tumors, they remain limited in curbing the metastatic and advanced NSCLC [3]. Key players of NSCLC progression, including noncoding RNA molecules and proteins, are under exploration at the moment [4–6]. Elucidating the precise roles of these molecular players will help to develop effectively targeted therapeutics.

Circular RNAs (circRNAs), as a covalently closed loop, are members of the noncoding RNA family commonly found in eukaryotic cells [7, 8]. In recent years, many researchers have devoted themselves to studying the role of circRNAs in various cancer diseases [9–11], including NSCLC [5, 12]. For example, circ_0001869 [13], circPTK2 [14], and circ_0000376 [15] have been studied to play a carcinogenic role in NSCLC. In addition, Chen et al. showed that circ_0078767 can inhibit the development of NSCLC by upregulating the expression of RASSF1 [16]. However, the mechanism of action of circ_0078767 in NSCLC has not been fully studied.

CircRNAs, as competitive endogenous RNAs (ceRNAs), can effectively regulate gene transcription through sponge microRNAs (miRNAs) [17]. Studies have shown that miRNA can interact with mRNA 3′UTR and exert negative regulation [18, 19]. Numerous studies have found that miR-665 plays a role in a variety of cancers. For example, Bai et al. showed that miR-665 regulates the progression of HCC under the targeting of circ_ABCC2 [20]. Wu et al.'s study found that miR-665 plays a tumor suppressive role in gastric cancer [21]. Xia et al. found that miR-665 played a carcinogenic role in NSCLC tissues and cells [22]. However, the mechanism of miR-665 in NSCLC has not been fully understood and is still worth studying.

Glutathione peroxidase 3 (GPX3) is an antioxidant enzyme, the only exocrine form of GPX, that protects cells from oxidative damage and regulates redox [23, 24]. Studies have shown that GPX3 plays a role in a variety of cancers [25], including ovarian cancer [26], breast cancer [27], and thyroid cancer [28]. Similarly, An et al. showed that GPX3 inhibited the proliferation of lung cancer cells [29]. In addition, several studies have shown that miRNA can inhibit the progression of NSCLC by regulating GPX3, such as miR-196a and miR-921 [30, 31]. However, it is still unclear whether GPX3 is regulated by miR-665 in NSCLC.

In this study, we discovered that circ_0078767 was highly expressed in NSCLC tissues and cells. Furthermore, we aimed to find that circ_0078767 can regulate NSCLC through a novel molecular mechanism of miR-665/GPX3 and come up with a novel idea for the cure of NSCLC.

## 2. Materials and Methods

### 2.1. Patients and Cell lines

40 cases of matched tumor tissues and adjacent normal tissues (ANT) were selected from patients with NSCLC admitted to Gansu Provincial Cancer Hospital. They did not receive any chemotherapy or radiation before the surgery. All patients signed patient informed consent and were approved by Gansu Provincial Cancer Hospital. The tissues samples were immediately frozen in liquid nitrogen and then stored in the refrigerator at -80°C until use.

Human bronchial epithelial cells (BEAS-2B) and lung cancer cell line (A549, HCC827 H1299) needed in this study were provided by American Type Culture Collection (ATCC; Manassas, VA, USA) and were all cultured in Dulbecco's Modified Eagle's Medium (DMEM; Invitrogen, Carlsbad, USA) containing 10% FBS in an incubator containing 5% CO_2_ at a constant temperature of 37°C.

### 2.2. Cell Transfection

RiboBio, Ltd. (Guangzhou, China) provided circ_0078767 small interfering RNA (si-circ_0078767#1, si-circ_0078767#2 and si-circ_0078767#3), and their controls (si-NC), miR-665 mimic/inhibitor (miR-665/anti-miR-665) and their control (miR-NC/anti-miR-NC), pcDNA-GPX3 (GPX3), and blank vector (pcDNA). A549 and HCC827 cells (1 × 10^5^ cells/well) were plated and cultured in 24-well plates 24 h before transfection. According to Lipofectamine 2000 (Promega, Madison, WI, USA) manufacturer's instructions, the above oligonucleotide (200 nM siRNA or 50 nM miRNA mimic/inhibitor) and plasmid (200 ng) constructs were transfected into A549 and HCC827 cells.

### 2.3. Quantitative Real-Time Polymerase Chain Reaction (qRT-PCR)

A549 and HCC827 cells were transfected for 48 h, and total RNA was extracted and reverse transcribed to cDNA using TRIzol reagents (Thermo Fisher, Waltham, MA, USA) and reverse transcription kit (Thermo Fisher), respectively, followed by qRT-PCR using SYBR Green qRT-PCR Mix (Takara, Shiga, Japan). GAPDH or U6 was used as internal reference for circRNA and miRNA, respectively, and the relative expression levels of circRNA and miRNA were calculated by 2^-*ΔΔ*CT^. The primer sequences and thermal conditions used in this experiment were shown in Supplement Table 1.

### 2.4. Western Blot Analysis

The total protein was extracted by adding 30 mg of removed fresh lung cancer tissue and transfected A549 and HCC827 cells to 250 *μ*L of RIPA lysate (Thermo Fisher), respectively, and the protein was quantified by BCA protein assay kit (Pierce; Rockford, IL, USA) and subjected to 12% SDS-PAGE electrophoresis and transferred to PVDF membranes. The membranes were subsequently blocked by milk and incubated with the following primary antibody: anti-*β*-actin (1 : 1,000, ab8226, Abcam, Cambridge, MA, USA), anti-hexokinase-2 (HK2; 1 : 1,000, ab227198, Abcam), anti-matrix metalloproteinase-9 (MMP9; 1 : 1,000, ab38898, Abcam), and anti-GPX3 (1 : 1,000, ab104448, Abcam), respectively. Finally, protein bands were visualized using the eyeECL Plus Kit (Beyotime, Shanghai, China).

### 2.5. Cellular Distribution Analysis

RNA extracted from A549 and HCC827 cells was processed using Cytoplasmic and Nuclear RNA Purification Kit (Norgen Biotek, Thorold, ON, Canada), followed by qRT-PCR to detect circ_0078767 in the cytoplasm and nucleus, with GAPDH and U6 as internal controls.

### 2.6. Colony Formation Assay

Transfected A549 cells were inoculated into 6-well plates with3 × 10^2^cells in each well and cultured conventionally for 2 weeks until visible cell colonies appeared, fixed with 4% paraformaldehyde and dyed with 0.5% crystal violet, and take pictures under a microscope.

### 2.7. Transwell Assay

The invasion and migration ability of A549 and HCC827 cells were analyzed by transwell chambers (Corning, NY, Madison, USA) with and without matrix gel coating in the upper layer, respectively. And serum-free medium was added to the upper layer of the transwell chamber, and medium containing 15% FBS was added to the lower layer as a chemical elicitor. 24 h later, the cells in the lower layer were counted by 1% crystalline violet staining, and a random field was selected under a high-powered microscope.

### 2.8. Glycolysis Analyses

Glycolysis was measured using a lactate assay kit and a glucose uptake colorimetric kit manufactured by Sigma-Aldrich Co., Ltd. (St. Louis, MO, USA) to assess lactate production and glucose consumption.

### 2.9. Dual-Luciferase Reporter Assay

To construct the dual luciferase reporter plasmid, wild-type (WT) and mutant (mutt) circ_0078767 or GPX3 3′UTR fragments including miR-665 binding sites or mutant sites were inserted into the pGL3-basic vector (Promega, Madison, WI) to form the WT-circ_0078767, MUT-circ_0078767, WT-GPX3 3′UTR, or MUT-GPX3 3′UTR reporter vectors. Subsequently, the above reporter plasmids were cotransfected into A549 and HCC827 cells with miR-665 mimic or miR-NC with Lipofectamine 2000, respectively. 48 h after transfection, luciferase activity was assessed by a Dual-Luciferase Reporter Assay Kit (GeneCopoeia, Rockville, MD, USA).

### 2.10. Xenograft Models

The BALB/c nude mice (6-8 weeks old) used in this study were purchased from Beijing Vidahe Laboratory Animal Technology Co., Ltd (Beijing, China), and animal experiments were performed according to the guidelines of the Animal Care Use Committee of Gansu Cancer Hospital. Sh-circ_0078767 and sh-NC provided by Ribobio, Ltd.were transfected into A549 cells (2 × 10^6^) and administered to BALB/c mice (*n* = 5) by subcutaneous injection. Tumor length and width were measured weekly after treatment, and tumor volume was calculated: volume = length/width^2^/2. Mice were neck broken and executed after 4 weeks, and subcutaneous tumors were removed and weighed.

### 2.11. Statistical Analysis

All experimental results in this study were analyzed using GraphPad Prism 7 software and presented as mean ± standard deviation (SD), and differences between groups were analyzed using Student's *t*-test. *P* < 0.05 was the difference is statistically significant.

## 3. Results

### 3.1. Circ_0078767 Was Low Expressed in NSCLC

**Figure 1:**
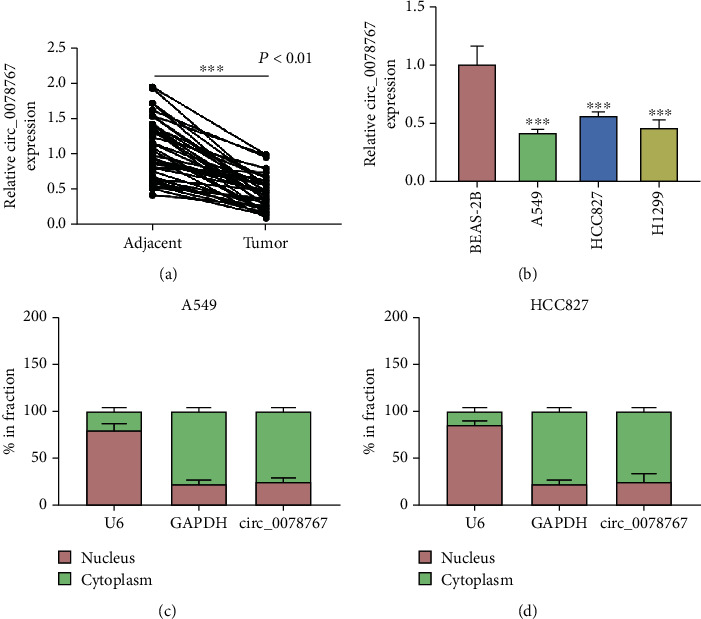
Circ_0078767 was upregulated in NSCLC tissues and cell lines. (a, b) The expression of circ_0078767 in NSCLC tissues (*n* = 40) and cells was tested by qRT-PCR. (c, d) The distribution of circ_0078767 in nucleus or cytoplasm was determined by qPCR. ^∗∗∗^*P* < 0.001.

We detected the expression of circ_0078767 in NSCLC tissues and cells by qRT-PCR, and the results showed that the expression of circ_0078767 was low in lung cancer tissues and cells (Figures 1(a) and 1(b)). Through cell distribution experiments, it was found that circ_0078767 was mostly distributed in the cytoplasm (Figures 1(c) and 1(d)).

### 3.2. Silencing circ_0078767 Promotes Proliferation, Migration, Invasion, and Glycolysis of NSCLC Cells

**Figure 2:**
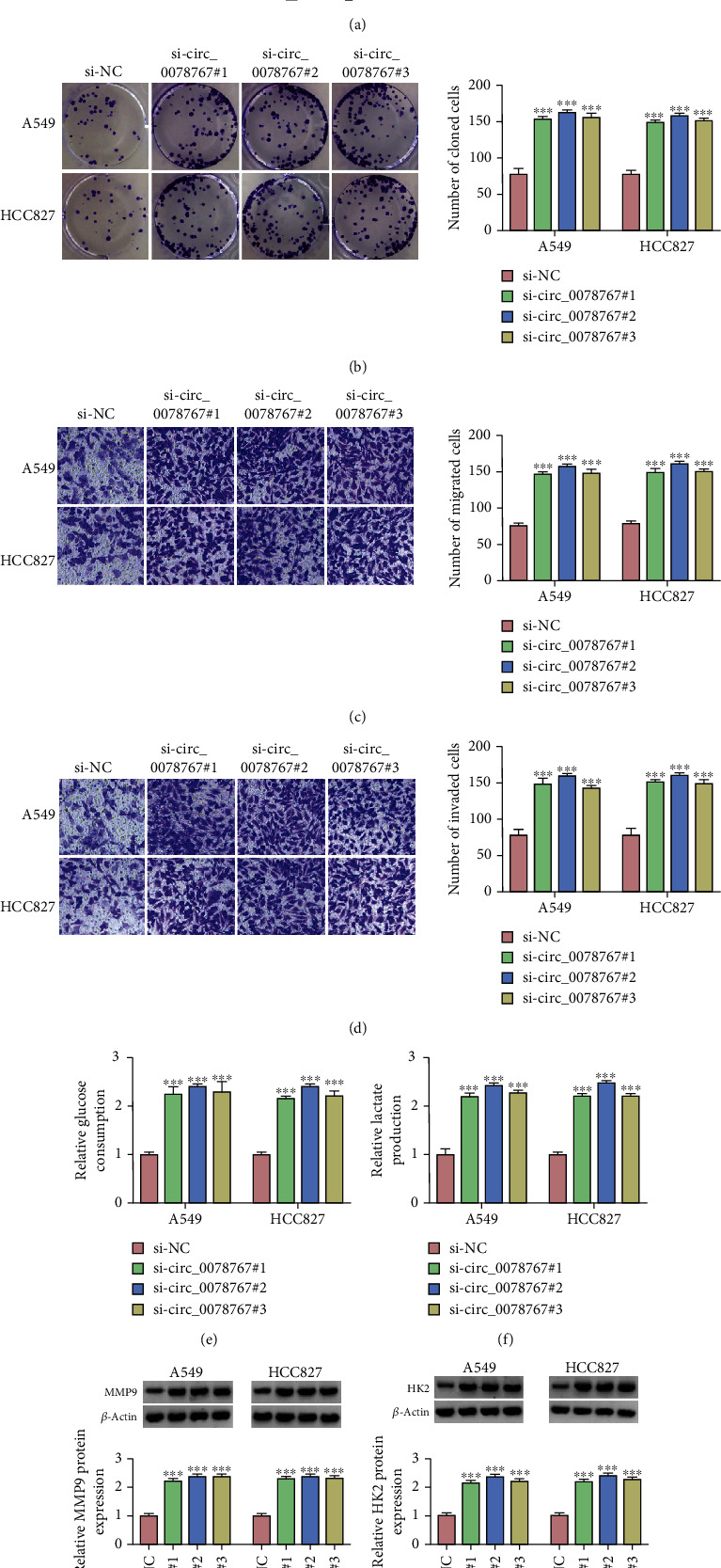
The functional roles of circ_0078767 on proliferation, migration, and invasion in NSCLC cells. (a) The expression level of circ_0078767 was determined by qRT-PCR assay in A549 and HCC827 cells transfected with si-NC or si-circ_0078767#1, si-circ_0078767#2, and si-circ_0078767#3. (b) Colony formation assay was used to assess proliferation of transfected A549 and HCC827 cells. (c, d) The role of circ_0078767 knockdown on cell migration and invasion was monitored by transwell assay. (e, f) Glucose consumption and lactate production were measured to assess glycolysis metabolism using commercial kits. (g, h) The protein expression level of MMP9 and HK2 was tested by Western blot analysis in transfected A549 and HCC827 cells. ^∗∗∗^*P* < 0.001.

We further investigated the effect of circ_0078767 on NSCLC cells by knocking down circ_0078767. First, si-circ_0078767#1, si-circ_0078767#2, and si-circ_0078767#3 were transfected into A549 and HCC827 cells, respectively. As shown in Figure 2(a), the knockdown efficiency of circ_0078767 in A549 and HCC827 cells was detected by qRT-PCR. Subsequently, the proliferation of transfected A549 and HCC827 cells was measured by colony formation assay, and the results showed that knockdown of circ_0078767 significantly promoted cell proliferation (Figure 2(b)). And the results of transwell test showed that the silencing of circ_0078767 significantly increased the migration and invasion rate of A549 and HCC827 cells (Figures 2(c) and 2(d)). Moreover, we examined glucose consumption and lactate production to assess glycolysis metabolism. The data showed that circ_0078767 knockdown significantly upregulated glucose consumption and lactate production in A549 and HCC827 cells (Figures 2(e) and 2(f)). Finally, Western blot results showed that the protein expression levels of MMP9 and HK2 in A549 and HCC827 cells were significantly increased after circ_0078767 knockdown (Figures 2(g) and 2(h)). In a word, silencing circ_0078767 promoted malignant behavior in NSCLC cells.

### 3.3. miR-665 Was a Target of circ_0078767

**Figure 3:**
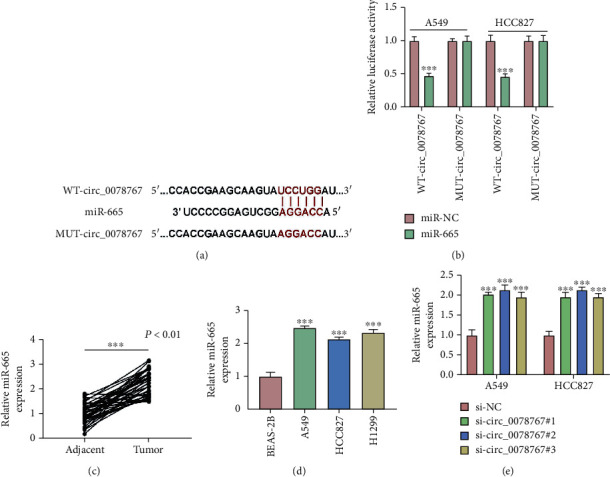
Circ_0078767 functions as a sponge of miR-665. (a) The complementary sequences between miR-665 and circ_0078767 were shown. (b) Dual-luciferase reporter assays were performed to confirm the association between miR-665 and circ_0078767. (c, d) The expression of miR-665 in NSCLC tissues (*n* = 40) and cells was tested by qRT-PCR. (e) The expression of miR-665 in NSCLC cells was tested by qRT-PCR. ^∗∗∗^*P* < 0.001.

We predicted that miR-665 was the target of circ_0078767 using the bioinformatic tool circinteractome (https://circinteractome.nia.nih.gov/mirna_target_sites.html). The binding sites of circ_0078767 and miR-665 are shown in Figure 3(a). Dual-luciferase reporter assay results showed that in A549 and HCC827 cells, the combined transfection of miR-665 and WT-circ_0078767 significantly inhibited luciferase activity, while the combined transfection of miR-665 and MUT-circ_0091579 showed no significant change (Figure 3(b)). Subsequently, we detected the expression of miR-665 in NSCLC tissues and cells by qRT-PCR, and the results showed that the expression of miR-665 in NSCLC tissues and cells was significantly increased compared with that in healthy tissues and cells (Figures 3(c) and 3(d)). And the qRT-PCR results showed that the expression level of miR-665 was significantly increased after circ_0078767 knockdown (Figure 3(e)). Therefore, the above data show that miR-665 was the target of circ_0078767.

### 3.4. MiR-665 Inhibitors Reversed the Promoting Effects of circ_0078767 Knockdown on Proliferation, Migration Invasion, and Glycolysis of NSCLC Cells

**Figure 4:**
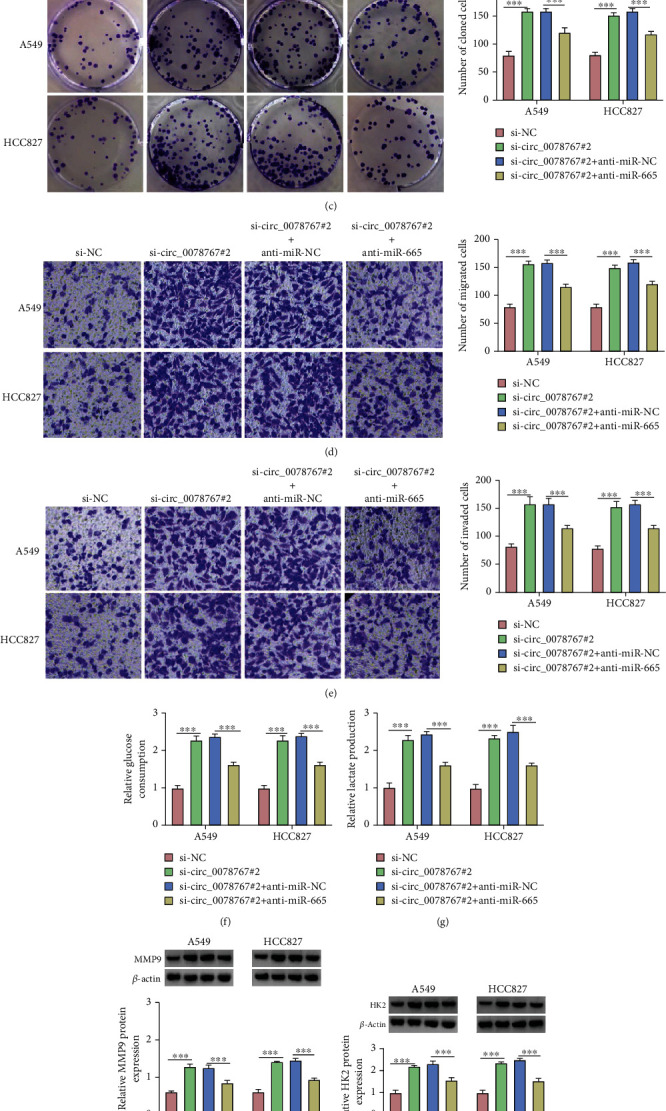
Circ_0078767 regulates the proliferation, migration, and invasion by targeting miR-665 in NSCLC cells. (a, b) The expression level of miR-665 was determined by qRT-PCR. (c) Colony formation assay was used to assess proliferation of transfected A549 and HCC827 cells. (g, h) Transwell assay was used to assess cell migration and invasion. (f, g) Glucose consumption and lactate production were measured to assess glycolysis metabolism using commercial kits. (h, i) The protein expression level of MMP9 and HK2 was tested by western blot analysis. ^∗∗∗^*P* < 0.001.

In order to explore the role of circ_0078767 and miR-665 in NSCLC cells, we first verified the overexpression and inhibition efficiency of miR-665 by qRT-PCR (Figure 4(a)), which was used for subsequent experiments. As shown in Figure 4(b), qRT-PCR results showed that the expression level of miR-665 was significantly increased after circ_0078767 silencing, while this expression was reversed after the addition of miR-665 inhibitor. Functionally, cotransfection with miR-665 inhibitors reversed A549 and HCC827 cell proliferation promoted by circ_0078767 knockdown (Figure 4(c)). Similarly, the increased rate of cell migration and invasion caused by si-circ_0078767#2 was also alleviated after the addition of anti-miR-665 (Figures 4(d) and 4(e)). Mechanically, the elevation of glucose consumption and lactate production caused by knocking down circ_0078767 was restored by transfection of anti-miR-665 (Figures 4(f) and 4(g)). Finally, Western blot results showed that the elevated MMP9 and HK2 protein levels induced by circ_0078767 knockdown were recovered by transfection of anti-miR-665 (Figures 4(h) and 4(i)). In short, these data suggest that silencing circ_0078767 promotes proliferation, migration, invasion, and glycolysis of NSCLC cells by upregulating miR-665.

### 3.5. MiR-665 Could Bind to GPX3 in NSCLC cells

**Figure 5:**
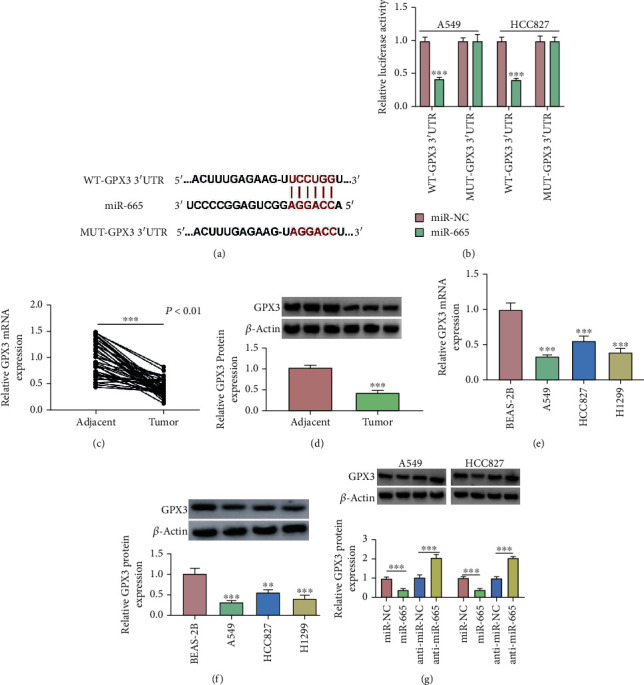
GPX3 is the direct target of miR-665. (a) The complementary sequences between miR-665 and GPX3 were shown. (b) Dual-luciferase reporter assays were performed to confirm the association between miR-665 and GPX3 3′UTR. (c, e) The expression of GPX3 in NSCLC tissues (*n* = 40) and cells was tested by qRT-PCR. (d, f) Western blot examined the protein level of GPX3 in NSCLC tissues (*n* = 40) and cells. (g) Western blot was used to detect the expression of GPX3 in transfected A549 and HCC827 cells. ^∗∗∗^*P* < 0.001.

We predicted that GPX3 was the target of miR-665 using the bioinformatic tool circinteractome (https://circinteractome.nia.nih.gov/mirna_target_sites.html). The binding sites of GPX3 and miR-665 are shown in Figure 5(a). Dual-luciferase reporter assay results showed that in A549 and HCC827 cells, the combined transfection of miR-665 and WT-GPX3 3′UTR significantly inhibited luciferase activity, while the combined transfection of miR-665 and MUT-GPX3 3′UTR showed no significant change (Figure 5(b)). Subsequently, we detected the expression of GPX3 in NSCLC tissues and cells by qRT-PCR and Western blot, and the results showed that the expression of GPX3 in NSCLC tissues and cells was significantly decreased compared with that in healthy tissues and cells (Figures 5(c)–5(f)). Moreover, the data of qRT-PCR and western blot showed that the expression of GPX3 was significantly decreased at both mRNA and protein by overexpression of miR-665, while the expression of GPX3 was significantly increased after the inhibition of miR-665 (Figure 5(g) and Supplement Figure 1A). Therefore, the above data show that GPX3 was the target of miR-665.

### 3.6. The GPX3 Overexpression Reversed the Promoting Effects of miR-665 on Proliferation, Migration, and Invasion of NSCLC Cells

**Figure 6:**
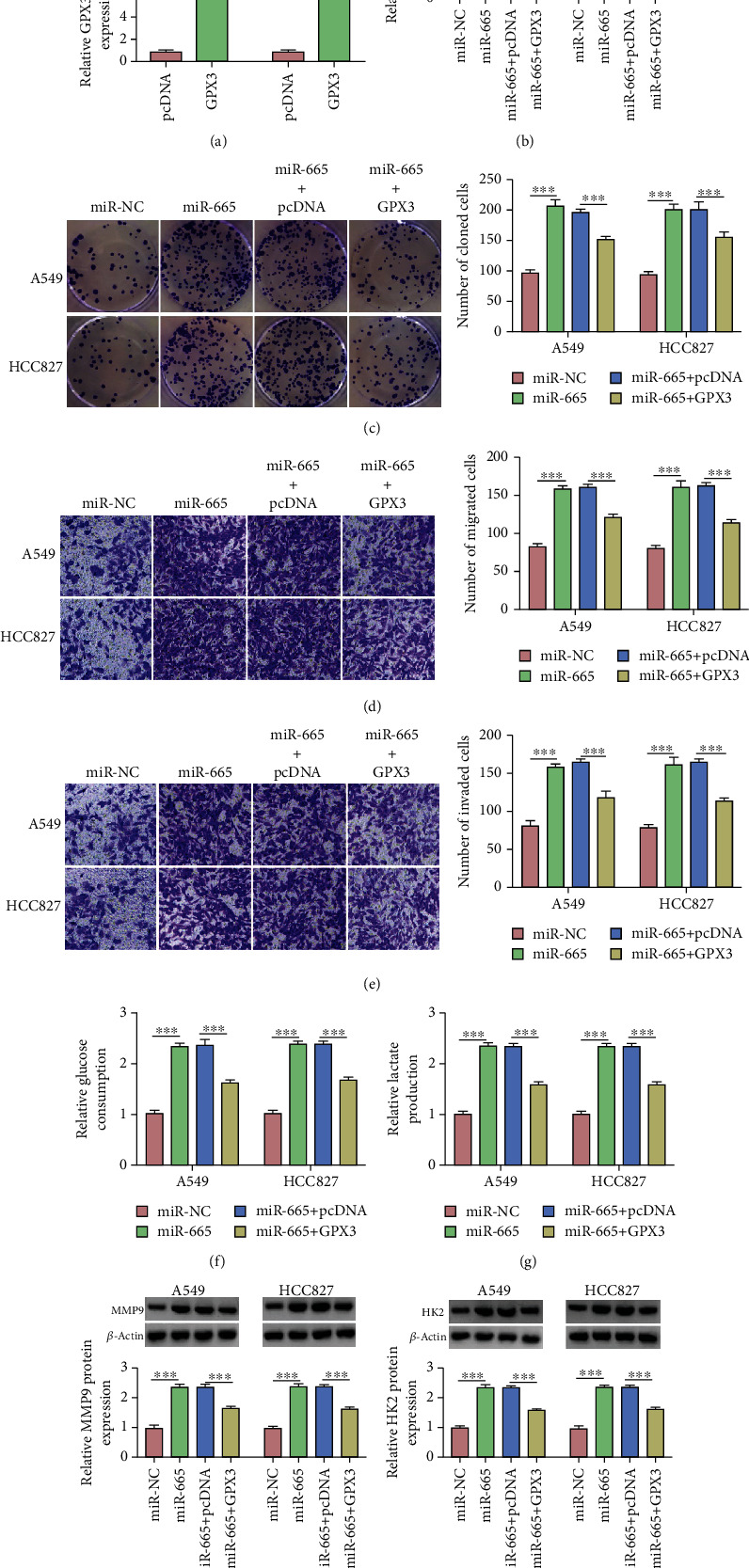
GPX3 regulates the proliferation, migration, and invasion by targeting miR-665 in NSCLC cells. (a, b) The protein level of GPX3 was determined by Western blot. (c) Colony formation assay was used to assess proliferation of transfected A549 and HCC827 cells. (d, e) Transwell assay was used to assess cell migration and invasion. (f, g) Glucose consumption and lactate production were measured to assess glycolysis metabolism using commercial kits. (h, i) The protein level of MMP9 and HK2 was detected by Western blot. ^∗∗∗^*P* < 0.001.

In order to further explore the role of GPX3 and miR-665 in NSCLC cells, we first confirmed the transfection efficiency of GPX3 in A549 and HCC827 cells by qRT-PCR and Western blot (Figure 6(a) and Supplement Figure 1B). Subsequently, the combined complement experiment showed that the overexpression of miR-665 could significantly reduce the expression of GPX3, while the expression level of GPX3 was effectively restored after cotransfection of the GPX3 expression plasmid (Figure 6(b) and Supplement Figure 1C). Functionally, cotransfection with the overexpression of GPX3 reversed A549 and HCC827 cell proliferation promoted by miR-665 (Figure 6(c)). Similarly, the increased rate of cell migration and invasion caused by the overexpression of miR-665 was also alleviated after the addition of GPX3 (Figures 6(d) and 6(e)). Mechanically, the elevation of glucose consumption and lactate production caused by the overexpression of miR-665 was restored by transfection of the overexpression of GPX3 (Figures 6(f) and 6(g)). Finally, Western blot results showed that the elevated MMP9 and HK2 protein levels induced by the overexpression of miR-665 were recovered by transfection of the overexpression of GPX3 (Figures 6(h) and 6(i)). In conclusion, these data suggest that abnormal upregulation of miR-665 promotes proliferation, migration, and invasion of NSCLC cells by inhibiting the GPX3 expression.

### 3.7. Validation of circ_0078767/miR-665/GPX3 Axis in NSCLC Cells

**Figure 7:**
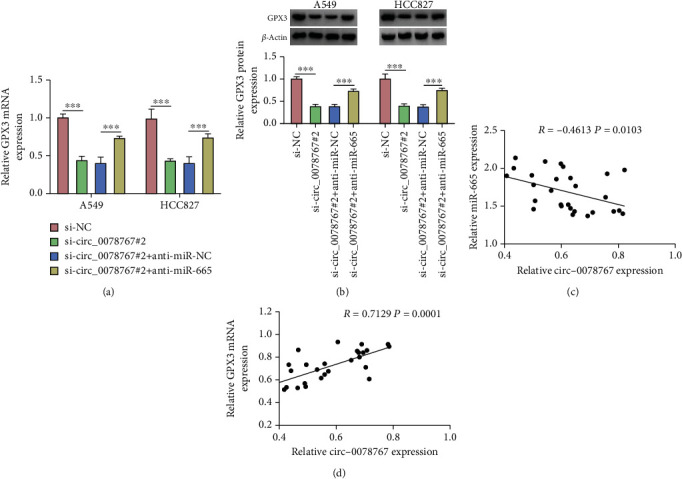
Circ_0078767 regulates the role of GPX3 via miR-665 in NSCLC. (a) The expression level of GPX3 was determined by qRT-PCR. (b) The protein level of GPX3 was determined by Western blot. (c, d) Pearson's correlation analysis. ^∗∗∗^*P* < 0.001.

In summary, our aim is to verify the molecular regulatory mechanism between circ_0078767/miR-665/GPX3. QRT-PCR results showed that downregulation of circ_0078767 reduced GPX3 protein level in NSCLC cells, while addition of anti-miR-665 restored GPX3 protein level in NSCLC cells (Figure 7(a)). Similarly, Western blot analysis of GPX3 also confirmed this result (Figure 7(b)). As shown in Figures 7(c) and 7(d), Pearson's correlation analysis results showed that circ_0078767 was negatively correlated with miR-665, while positively correlated with GPX3. In general, circ_0078767 regulates the GPX3 expression by sponging miR-665 in NSCLC cells.

### 3.8. Circ_0078767 Knockdown Promoted Tumor Growth In Vivo

**Figure 8:**
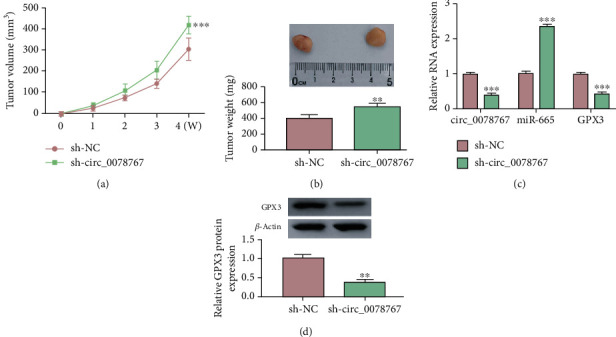
Circ_0078767 knockdown promoted tumor growth in vivo. (a, b) Tumor volume and weight after circ_0078767 knockdown in vivo. (c) Relative expression levels of circ_0078767, miR-665, and GPX3 in xenografts were detected by qRT-PCR. (d) GPX3 protein level tested by western blot in xenografts. ^∗∗∗^*P* < 0.001.

We observed the effect of sh-circ_0078767 on NSCLC tumors through xenograft experiment in nude mice in vivo, and the results showed that circ_0078767 knockdown could significantly increase tumor volume and weight (Figures 8(a) and 8(b)). In addition, qRT-PCR detection data showed that the expression level of circ_0078767 and GPX3 was significantly decreased in the transplanted tumor with circ_0078767 knockdown, while the level of miR-665 was significantly increased (Figure 8(c)). Finally, the expression of GPX3 in the transplanted tumor was significantly reduced by Western blot analysis (Figure 8(d)). In conclusion, knockdown of circ_0078767 can enhance tumor growth.

## 4. Discussion

Numerous studies have shown that circRNAs can be involved in the progression of a variety of cancers. In this study, we confirmed the low expression of circ_0078767 in lung cancer patients and lung cancer cell lines by qRT-PCR. We also confirmed the inhibitory effect of circ_0078767 on tumor growth in vivo by constructing a xenotransplantation model in nude mice. Warburg effects believed that glycolysis is the main source of energy metabolism in cancer cells, which converts glucose into lactic acid through glycolysis, and ATP produced by lactic acid is used to maintain the growth and progression of tumors [32]. The role of circRNAs in regulating glycolysis in cancer disease has been extensively studied in recent years [33]. For example, downregulation of circSEC31A or circ_0000735 can inhibit glycolytic metabolism in NSCLC cells [34, 35]. In this study, knocking down circ_0078767 significantly promoted the proliferation, migration, and invasion of NSCLC cells. In addition, silencing circ_0078767 promoted glucose consumption and lactate production and significantly upregulated the expression of HK2 protein, suggesting that downregulation of circ_0078767 promoted glycolytic metabolism. Our results are consistent with the low expression of circ_0078767 in NSCLC studied by Chen et al. [16], suggesting that circ_0078767 may be a key target for the treatment of NSCLC.

According to the above results, we understood the mechanism of circ_0078767 in NSCLC. Since circRNAs can adsorb other miRNAs and play a regulatory role, then, we found that miR-665 is a target gene of circ_0078767. A series of complement experiments were designed to explore whether it can regulate NSCLC. We confirmed that miR-665 was highly expressed in NSCLC tissue cells and had a negative regulatory relationship with circ_0078767. In addition, downregulation of miR-665 significantly inhibited malignant behavior and glycolytic metabolism of NSCLC cells promoted by circ_0078767 silencing. Our results are consistent with those of previous studies [22].

As a tumor suppressor gene, GPX3 may be involved in the occurrence of a variety of cancer diseases [25]. It has been reported that in lung cancer cells, GPX3, as a redox regulator, inhibits the production of ROS and blocks the G2/M phase of the cell cycle, thereby inhibiting the proliferation of lung cancer cells [29]. In addition, GPX3 was found to be the target gene of several miRNAs in NSCLC [28, 30]. Similarly, our results revealed that GPX3 was a downstream target of miR-665, and that aberrant upregulation of miR-665 inhibited the GPX3 expression. In terms of function, the overexpression of GPX3 restored the malignant behavior as well as glycolysis of A549 and HCC827 cells promoted by the miR-665 overexpression. And we made clear that downregulated circ_0078767 remarkably attenuates the GPX3 expression by upregulating miR-665, indicating that circ_0078767 modulated the NSCLC cell behavior via miR-665/GPX3 pathway.

## 5. Conclusion

In conclusion, we indicated that circ_0078767 was diminished in NSCLC tissues and cells, which regulated the progression of NSCLC by modulating the miR-665/GPX3 axis. These data provided evidences that upregulation of circ_0078767 might be used as a treatment for NSCLC.

## Data Availability

The data used to support the findings of this study are available from the corresponding author upon request.

## Additional Points

*Highlight*. (1) Circ_0078767 was downregulated in NSCLC tissues and cells, and circ_0078767 knockdown could promote the proliferation, migration, and invasion of NSCLC cells. (2) MiR-665 was the target of circ_0078767 in NSCLC cells. (3) GPX3 was the target of miR-665, and the overexpression of GPX3 can inhibit the promotion of miR-665 on malignant behavior of cells.

## Supplementary Materials

Supplementary 1 Supplement Table 1: primer sequences used for qRT-PCR.

Supplementary 2 Supplement Figure 1: expression of GPX3 mRNA in transfected A549 and HCC827 cells. (a)–(c) qRT-PCR of the GPX3 mRNA expression in cells transfected as indicated. ^∗∗∗^*P* < 0.001.
