# Supplementary material for: Circ_0078767 Inhibits the Progression of Non-Small-Cell Lung Cancer by Regulating the GPX3 Expression by Adsorbing miR-665
Source: Int J Genomics. 2022 Mar 17;2022:6361256. doi: 10.1155/2022/6361256 (PMC8948607; doi:10.1155/2022/6361256)
Supplement: Supplementary 1 — Supplement Table 1: primer sequences used for qRT-PCR. [file 6361256.f1.docx]

**Supplement Table 1. Primers sequences used for qRT-PCR**

| **Name** |  | **Primers for PCR (5’-3’)** |
| --- | --- | --- |
| circ_0078767 | Forward | GCCTAGCTGTCAAGGAGTGG |
|  | Reverse | GGATCTAGAGATGCGCCAAC |
| GPX3 | Forward | TGGTCATTCTGGGCTTTCCC |
|  | Reverse | CCAGAAGAGGCGGTCAGATG |
| miR-665 | Forward | GTATGAACCAGGAGGCTGAG |
|  | Reverse | CAGTGCGTGTCGTGGAGT |
| U6 | Forward | CTCGCTTCGGCAGCACATATACT |
|  | Reverse | ACGCTTCACGAATTTGCGTGTC |
| GAPDH | Forward | GGAGCGAGATCCCTCCAAAAT |
|  | Reverse | GGCTGTTGTCATACTTCTCATGG |
| Thermal conditions | 95°C, 10 min; 95°C, 15 seconds for 40 cycles; 60°C, 1 min | |
