# Supplementary figures and images for: Circ_0078767 Inhibits the Progression of Non-Small-Cell Lung Cancer by Regulating the GPX3 Expression by Adsorbing miR-665
Source: Int J Genomics. 2022 Mar 17;2022:6361256. doi: 10.1155/2022/6361256 (PMC8948607; doi:10.1155/2022/6361256)

**A**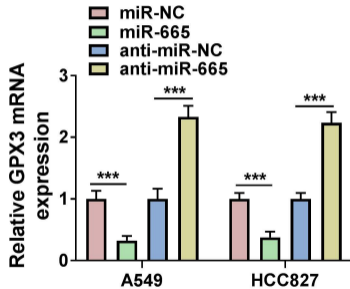**B**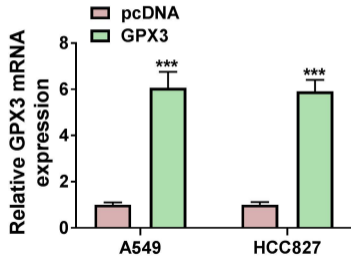**C**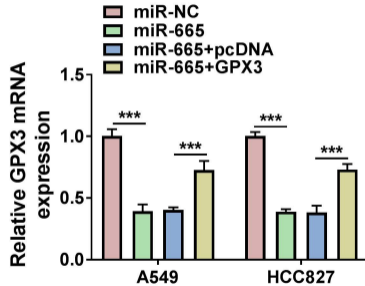

Supplement: Supplementary 2 — Supplement Figure 1: expression of GPX3 mRNA in transfected A549 and HCC827 cells. (a)–(c) qRT-PCR of the GPX3 mRNA expression in cells transfected as indicated. ∗∗∗P < 0.001. [file 6361256.f2.pdf]
